# Taking the Stem Cell Debate to the Public

**DOI:** 10.1371/journal.pbio.0020188

**Published:** 2004-06-15

**Authors:** Leonard I Zon, Laurie Zoloth, Suzanne Kadereit

## Abstract

In response to the Blackburn and Rowley essay on the President's Council on Bioethics, several thought-provoking opinions on ethical challenges in biomedical research are expressed by prominent stakeholders

In their essay in the April 2004 issue of *PLoS Biology*, Elizabeth Blackburn and Janet Rowley (2004), two distinguished cellular biologists and members of the President's Council on Bioethics, strongly question the scientific foundation of two reports from the Council (President's Council on Bioethics 2003, 2004). The Council on Bioethics was formed by executive order “to advise the President on bioethical issues that may emerge as a consequence of advances in biomedical science and technology.” An open discussion between ethicists and scientists is critical to the advisory system. The recent administrative dismissal of Dr. Blackburn from the Council is very alarming. By stacking the deck with conservative opinions, and not accurately discussing the scientific issues, the Bioethics Council has become irrelevant to the scientific community and presents a jaundiced view to the public.

Stem cell research and its applications have the potential to revolutionize human health care. Recent polls show support for embryonic stem cell research, even with conservative voters. The public, as the major benefactor of biomedical research and the target population of beneficial clinical advances, has the right to a fact-based discussion of the science regarding stem cells. It is therefore time that the debate on stem cell research, with its risks and benefits, be taken to the public. A debate on stem cell research restricted to the President's Council on Bioethics is a disservice to the public.

Nearly three decades ago, the advent of recombinant DNA technology and in vitro fertilization (IVF) techniques, raised similar concerns regarding research. Contrary to apprehensive expectations, recombinant DNA technology has boosted enormous advances in the health care and pharmaceutical industry. IVF evolved to be a widely accepted, safe medical procedure, with over one million healthy babies born by IVF and related treatments. Similarly, once stem cells are successfully used in the clinic, most of today's political and ethical issues will evaporate.

The International Society for Stem Cell Research (ISSCR), a society whose membership encompasses the bulk of the stem cell research brain trust, holds the position that research on both adult and embryonic stem cells will guarantee the fastest progress in scientific discovery and clinical advances. The ISSCR also strongly opposes reproductive cloning and supports the National Academy of Science's proposal to develop voluntary guidelines to encourage responsible practices in human embryonic stem cell research.

One of the original recommendations of the President's Council on Bioethics was a four-year moratorium on stem cell research. The purpose of this moratorium was theoretically to open a large, national discourse on the topic of stem cell research, a debate intended to bring all sides into thoughtful reflection on the issue. To that end, the ISSCR has repeatedly and consistently offered an open forum for all sides in the debate at our conferences, and has carefully offered invitations to join our society and to speak at our annual meeting to members of the President's Council, including colleagues whose opposition to stem cell research has been clear. None have accepted. Dr. Kass, in particular, has received several direct appeals but has turned down every such opportunity to make his case to the researchers who arguably are his discourse partners, from whom he could learn much, and whom he should be actively engaged in teaching. It is tragic that voices of dissent and debate are stilled, for it is this very quality of open debate that is at the heart of both the scientific method and an ethically directed American democracy—surely a goal that we all share.
